# Effects of Ultra-Processed Foods and Food Additives on Disease Activity in Adults with Inflammatory Bowel Disease: A Scoping Review

**DOI:** 10.3390/jcm14217798

**Published:** 2025-11-03

**Authors:** Andrea Soublette Figuera, Sara Alessa, Carolyne Brien, Mary Hendrickson, Popi Kasvis, Talat Bessissow

**Affiliations:** 1 School of Human Nutrition, McGill University, Montreal, QC H9X 3V9, Canada; andrea.soublettefiguera@mail.mcgill.ca (A.S.F.); mary.hendrickson@mcgill.ca (M.H.); kalliopi.kasvis@mcgill.ca (P.K.); 2 Division of Internal Medicine, McGill University Health Centre (MUHC), Montreal, QC H4A 0B1, Canada; sara.alessa@mail.mcgill.ca; 3 Department of Clinical Nutrition, McGill University Health Centre (MUHC), Montreal, QC H4A 0B1, Canada; carolyne.brien@muhc.mcgill.ca; 4 Division of Gastroenterology and Hepatology, McGill University Health Centre (MUHC), Montreal, QC H4A 0B1, Canada

**Keywords:** inflammatory bowel disease, Crohn’s disease, ulcerative colitis, ultra-processed food, food additives

## Abstract

**Background/Objectives**: This scoping review aims to identify and map out the current state of research on the relationship between ultra-processed foods (UPF) intake and disease activity and symptoms in adults with inflammatory bowel disease (IBD). **Methods**: A search strategy was developed using key terms, and the search was conducted through the PubMed, Medline, Embase, CINAHL, Web of Science, The Cochrane Library, Google Scholar, and ProQuest Theses and Dissertations databases to identify relevant studies. Data were comprehensively extracted, analyzed, and synthesized. **Results**: A total of 18 studies were included in the review: 7 on UPF and 11 on food additives. Most prospective cohort and cross-sectional studies suggested a positive association between UPF intake and IBD activity, measured using disease activity indexes and fecal calprotectin (FC) as an inflammatory marker. Trials excluding food additives from the diet provided inconclusive evidence regarding their effectiveness in reducing IBD symptoms or disease activity. **Conclusions**: Current evidence suggests potential links between UPF and food additive intake and IBD outcomes. Yet, heterogeneity in UPF definitions, variability in additive formulations, and the lack of standardized dietary assessment methods limit definitive conclusions. Future studies should integrate standardized exposure assessment methods with validated outcome measures to better clarify the role of UPF and food additives on IBD trajectory.

## 1. Introduction

Inflammatory bowel disease (IBD), which includes Crohn’s disease (CD) and ulcerative colitis (UC), is a group of chronic immune-mediated conditions characterized by relapsing inflammation of the gastrointestinal (GI) tract. While genetic and immune factors are central to disease development, environmental and dietary factors are increasingly recognized as significant modulators of disease activity and symptom severity [1]. Over the past few decades, the global incidence and prevalence of IBD have risen markedly. This rising incidence across diverse geographic regions mirrors global changes in environmental exposure, lifestyle, and dietary habits associated with industrialization [2]. Among these environmental factors, ultra-processed foods (UPF) and specific food additives have been linked to an increased risk of IBD development [3].

**Figure 1:**
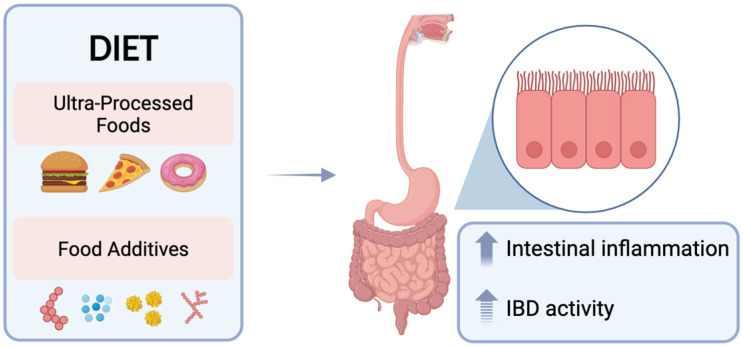
Framework illustrating the proposed relationship between dietary intake and inflammatory bowel disease (IBD). Dietary patterns rich in ultra-processed foods and food additives may influence gastrointestinal physiology and promote intestinal inflammation, which in turn could contribute to increased IBD activity. Dashed arrow denotes a potential or hypothesized association.

Although the association between UPF consumption and risk of IBD development is supported by prospective data [3,4,5], the impact of UPF on disease activity and frequency of flares remains unclear. Some studies suggest a positive association between UPF intake and increased risk of disease flare-ups, particularly in UC [6]; however, the findings often vary depending on the food classification system used, which may capture different aspects of food processing and composition, and overall nutrient profile (Figure 1). Additionally, there is limited consensus on which additives or processing methods may be most detrimental [7] and whether specific patient populations are more susceptible to their effects due to socioeconomic factors [8,9,10].

Assessing UPF and food additives intake in both research and clinical contexts remains challenging due to inconsistent food classification systems that vary in defining formulations, processing methods, and industrial ingredients [11,12,13,14,15]. These include the NOVA system, the International Agency for Research on Cancer (IARC) approach, the International Food Information Council (IFIC) model, and the University of North Carolina (UNC) framework, among others [16,17,18]. Emerging food classification systems, such as the WISEcode-UPF™, aim to standardize definitions. Despite these challenges, increasing evidence links UPF consumption to a wide range of adverse health outcomes [19,20], including the development and progression of IBD.

Given the increasing prevalence of IBD globally [1,21] and the parallel rise in UPF consumption [22], understanding how these foods influence disease course is of high clinical relevance. A better grasp of the existing evidence can inform dietary recommendations, patient counseling, and public health policy aimed at reducing preventable flares and improving quality of life in this population.

This scoping review aims to look at and map out the current state of research on the relationship between UPF intake and disease activity and symptoms in adults with IBD. Specifically, we will describe the various definitions and classifications of UPFs used in the literature. Second, we will summarize evidence on the impact of UPFs and food additives on disease flare-ups and GI symptoms in IBD patients. Lastly, we will identify gaps in knowledge and directions for future research.

## 2. Materials and Methods

To address our research question, a scoping review was conducted in accordance with the Preferred Reporting Items for Systematic Reviews and Meta-Analysis (PRISMA) checklist (Supplementary Material) [23]. A thorough search protocol was implemented to retrieve relevant studies across a wide range of electronic databases, including Medline, Embase, CINAHL, PubMed, Google Scholar, Web of Science, the Cochrane Library, and ProQuest Dissertations and Theses.

A preliminary search was conducted to identify relevant keywords and search terms. The final search strategy was developed in collaboration with an experienced librarian at McGill University Health Centre (MUHC) to ensure a comprehensive and inclusive approach that minimized the risk of overlooking relevant studies. The search was conducted in June 2025, incorporating a combination of Medical Subject Headings (MeSH) terms and keywords, which were tailored to each specific database. The full search strategy for Medline is included in Appendix A.

The search was executed on 25 June 2025. All retrieved citations were imported into Rayyan (version 1.6.5), an online platform designed to support management of systematic and scoping reviews [24]. This tool was also used to identify and remove duplicate records efficiently. Following duplicate elimination, three independent reviewers (A.S.F., M.H., P.K.) screened all titles and abstracts against the inclusion and exclusion criteria (Table 1). Full-text screening was later conducted for potentially eligible studies (A.S.F., S.A.). Any conflicts arising during the study selection process were resolved through discussion to reach consensus. The references of included studies were screened to identify additional relevant studies and to strengthen the search process.

For each included study, the following data was extracted: first author, year of publication, country, sample size, study population, study design, study funding, exposures and interventions, UPF classification system used (Table 2), food additives studied, outcome measures, results (impact on disease activity and symptoms), and conclusions. To facilitate the summary of results and their interpretation, findings of studies on UPF were grouped into those with positive associations and those with null or inverse associations. On the other hand, studies on food additives were grouped according to the specific food additive they investigated.

## 3. Results

**Figure 2:**
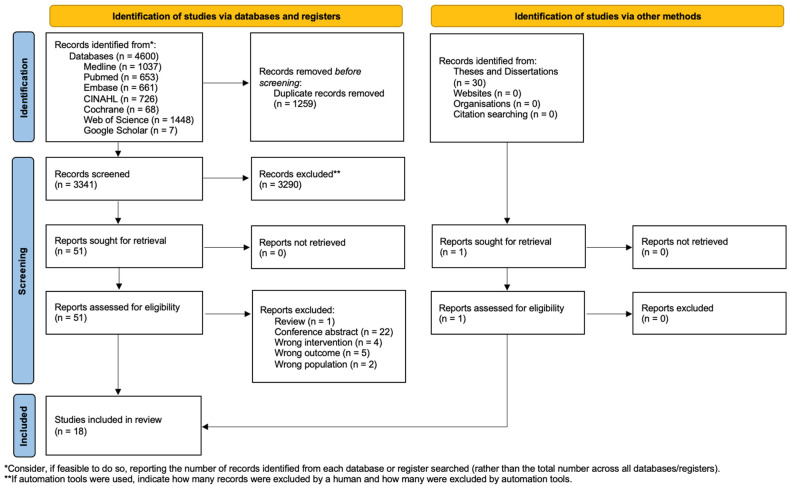
Prisma 2020 flow diagram of evaluated studies.

The initial database search identified 4630 records. After removing 1255 duplicates, 3375 records remained for title and abstract screening. Following initial screening, 3323 studies were excluded, and 52 studies were retrieved for full-text screening. Of these, 34 studies were excluded based on the eligibility criteria, and 18 studies were included in the qualitative synthesis. The study selection process is illustrated in the PRISMA flowchart (Figure 2) [23].

Among the 18 included studies, 7 investigated UPF exposure or interventions, while 11 focused on food additive exposure or interventions. The characteristics of studies examining UPF are presented in Table 3, and those addressing food additives are shown in Table 4.

### 3.1. UPF and Disease Activity in IBD

Seven studies published between 2023 and 2025 examined the effects of UPF intake on disease activity or IBD symptoms. These included four prospective cohort studies [6,25,29,30], one non-randomized trial [26], and two cross-sectional studies [27,28].

#### 3.1.1. Definition of UPF and Classification Systems

Out of the seven studies, five used the Nova food classification [6,25,28,29,30], one used an author-established classification [26], and one used the short questionnaire of highly processed foods (sQ-HPF) to identify highly processed foods (HPF) [27]. Notably, Pueschel et al. distinguish between the levels of processing in HPF and UPF, describing HPF as a broader category of foods including products such as butter, cream, honey, homemade desserts and homemade fried foods, which may be considered UPF in some classification systems [27]. Conversely, they define UPF as energy-dense foods with suboptimal micronutrient content and often containing food additives [27]. Despite this distinction, they recognize that both HPF and UPF can be high in fat, sugar, and sodium. While Pueschel et al. propose a difference, the overlap in definitions and the lack of a standardized classification system suggest that foods identified as HPF in their study could reasonably meet the criteria for UPF in other systems. Thus, to ensure comprehensive coverage and prevent the exclusion of relevant data, this scoping review includes their study and will treat HPF and UPF as interchangeable terms in this context.

#### 3.1.2. Positive Associations

Three studies reported positive associations between UPF intake and disease activity. Sarbagili-Shabat et al. followed a cohort of CD patients in remission and found that those consuming >3.6 servings/day of UPF (Nova 4) had a higher probability of relapse, defined as a Harvey Bradshaw Index (HBI) ≥ 5, (33.7% vs. 15.1%, *p* = 0.032), which was confirmed in a Cox regression model (HR = 3.86; 95% CI 1.30–11.47) [29]. Ultra-processed breads, pastries, starches, oils, and spreads were the food categories most strongly associated with relapse risk. A cross-sectional study reported similar findings, where higher UPF intake was associated with active disease in CD (OR = 3.82), and particularly higher ultra-processed meat intake (OR = 4.45), followed by beverages, starches and pastries, and oils and spreads [28]. In contrast, Vagianos et al. reported a significant association between UPF intake and active disease in UC (*p* = 0.012), but not in CD [6]. In UC, greater Nova 4 food intake was associated with increased fecal calprotectin (FC) (*p* = 0.018), while lower Nova 1 intake was linked to significantly more disease activity and inflammation. These findings highlight the potential influence of certain food groups within Nova 4 on IBD activity.

#### 3.1.3. Null or Inverse Associations

Some studies did not support a clear association between UPF intake and disease activity. In a 1-year cohort study, Koppelman et al. found 15% of IBD patients in remission relapsed (C-reactive protein (CRP) ≥ 5 mg/L or FC ≥ 150 µg/g), and 40% of those with active disease achieved remission, but Nova 4 UPF intake did not differ between remission and active patients or over time [25]. Similarly, in a non-randomized trial, Nitescu et al. compared IBD patients following an exclusion diet (removing UPF as categorized by the authors) or a regular diet over six months [26]. Although 100% of the exclusion group and 95.7% in the control group maintained remission, the difference was not statistically significant, nor were the changes in FC between groups (*p* = 0.067). Moreover, cross-sectional findings by Pueschel et al. found that despite a higher intake of HPF in an IBD cohort compared to controls, there was no significant association between HPF intake (defined by the sQ-HPF) and FC or CRP [27].

In a 2-year prospective cohort, Stevens et al. assessed the predictive value of food groups on disease flares, measured by a combination of endoscopic, biochemical (FC, CRP), and clinical outcomes such as the HBI and the Simple Clinical Colitis Activity Index (SCCAI) [30]. Non-UPF items (Nova 1, 2, and 3), particularly legumes, red meat, and vegetables, were more strongly associated with flare risk, while UPF intake appeared inversely associated with flares, suggesting a potential protective effect. This challenges previous assumptions and suggests the role of UPF intake in IBD may be population- or context-specific.

### 3.2. Food Additives and Disease Activity in IBD

Twelve records published between 1997 and 2025 examined the effects of food additive consumption on disease activity or IBD symptoms. These included 3 RCTs [34,37,40], a randomized placebo-controlled trial [31,35], a randomized feeding trial [32], a randomized crossover trial [33], a prospective feasibility study [36], an interventional prospective study [40], a cross-sectional study [9], and a provocation trial [39]. These studies investigated a variety of food additives, including emulsifiers, thickeners, food colorants, stabilizers, sweeteners, preservatives, and fat substitutes.

#### 3.2.1. Emulsifiers and Thickeners

Several studies have investigated the effect of emulsifiers, particularly carrageenan, on IBD. In a randomized placebo-controlled trial, Bhattacharyya et al. assessed carrageenan exposure in adults with UC in remission [31]. Subjects were instructed to follow a carrageenan-free diet for one year before being randomized to receive either 100 mg of carrageenan or dextrose capsules. The carrageenan group experienced significantly higher SCCAI scores compared to placebo (4.20 ± 3.70 vs. 0.86 ± 1.46, *p* = 0.05) and an increased risk of relapse (*p* = 0.046), with relapses occurring at 5, 32, and 42 weeks. Moreover, FC and interleukin-6 increased in the carrageenan group. These findings suggest that carrageenan may contribute to an increase in UC disease activity and risk of relapse, which may involve inflammatory pathways.

In contrast, a randomized crossover trial in adults with UC in remission found no significant effect of carrageenan on disease activity [33]. Subjects consumed dietary carrageenan and a placebo oat fiber product for 7 days each, with a 14-day washout period. No significant differences between periods were observed in SCCAI scores (*p* = 0.250) or in inflammatory markers (FC and CRP). These results may be related to the short-term nature of the exposure or to a limited sample size (*n* = 7), meaning further investigation is required.

Moreover, in a doctoral thesis submitted in 2021, Sandall examined the effects of a low-emulsifier diet (LED) against an emulsifier-containing control diet in a randomized controlled trial (RCT) of 10 adults with active CD [34]. Despite four participants experiencing improvements, the preliminary findings showed no significant group-level changes in CDAI scores from baseline to the end of the trial (*p* = 0.091), suggesting limited statistical power to detect significance. More recently, Fitzpatrick et al. compared an LED to a high-emulsifier diet (HED) in CD patients, showing that 9/12 subjects in the HED group and 7/12 in the LED group (*p* = 0.67) achieved clinical remission after 4 weeks [32]. No significant differences were found in inflammatory markers or gastrointestinal (GI) symptoms. Collectively, these findings suggest that while long-term carrageenan exposure may influence UC, short-term reductions in emulsifier intake may not contribute to improvements in CD activity.

#### 3.2.2. Colorants and Microparticles

Titanium dioxide (TiO2) and particulate silicates (Psil) are microparticles added to food as whitening and anti-caking agents. To examine their role in IBD, Lomer et al. conducted a randomized trial in patients with active CD, assigning them to 4 treatment groups: low calcium low microparticles (LCLM), low calcium normal microparticles (LCNM), normal calcium low microparticles (NCLM), and normal calcium normal microparticles (NCNM) [35]. Results showed no significant changes in CDAI, CRP, FC or intestinal permeability at 16 weeks or at one-year follow-up, suggesting that lowering microparticle intake does not improve CD activity or inflammatory markers.

#### 3.2.3. Whole-Diet Interventions Targeting Food Additives

Three studies from Australia explored the effects of whole-diet interventions that broadly restricted food additives in patients with IBD. These interventions varied in design but commonly aimed to reduce the exposure to food additives such as emulsifiers and preservatives and assessed the changes in IBD activity.

Day et al. evaluated the 4-SURE diet in adults with mild to moderate UC [36]. This four-week intervention aimed to reduce the intake of sulfur dietary components and food additives including carrageenan. Intake was assessed using food frequency questionnaires and 24 h recalls. Participants experienced statistically significant improvements in SCCAI scores (*p* = 0.02). Serum CRP levels did not change, but fecal calprotectin levels significantly decreased (median reduction by 131 µg/g, *p* = 0.04). These findings support the potential utility of additive restriction as an approach to controlling colonic inflammation.

On the other hand, a randomized study by Trakman et al. (The DELECTABLE Program) compared three dietary interventions: the Crohn’s Disease Exclusion Diet (CDED), the Ulcerative Colitis Diet (UCD), and a Whole Food Diet (WFD). The latter 2 were designed by the research team to exclude UPFs and food additives [38]. Study participants included patients with CD (*n* = 36), UC (*n* = 25), and microscopic colitis (*n* = 2). At baseline, diet choice was determined through shared decision making, taking into account disease characteristics, lifestyle, and patient preference. CDAI significantly decreased in the CDED (*p* = 0.023) and WFD (0.038) groups. CRP significantly reduced in the CDED arm (*p* = 0.043), while the partial Mayo score improved in the WFD group (*p* = 0.02). No statistically significant changes were observed in the UCD group, likely due to the small sample size.

An Australian RCT examined the efficacy of an anti-inflammatory diet in reducing disease activity in adults with IBD [37]. Fifty-eight patients, the majority with UC (75%), were randomized to either general healthy eating education or to the Modified Anti-Inflammatory Diet (IBD-MAID), which was designed to reduce the intake of food additives. Over a 16-week period, dietary intake was assessed using food diaries and food additive scores. CDAI decreased in both groups; however, there was no statistically significant difference between the groups, and no difference was observed in SCCAI. Moreover, a significant reduction in FC was observed in the intervention group, though the difference compared to the control group was not statistically significant.

#### 3.2.4. General Additive Exposure

Zorich et al. conducted one of the earliest randomized trials evaluating the impact of food additives on IBD by examining the effect of olestra, a fat substitute, on disease activity [40]. Subjects were randomized to receive either 20 g of olestra or conventional triglycerides in snack foods for 4 weeks. Relapse rates and reports of worsening symptoms were similar between both groups. Thus, the authors suggested the findings indicate olestra showed no detrimental effects on disease activity or general health.

In a cross-sectional context, Trakman et al. assessed dietary intake and exposure to processed food and food additives among CD patients in Australia, Hong Kong, and mainland China [9]. Using food diaries and dietary questionnaires, the authors found a higher intake of food additives in CD patients compared to controls across multiple regions (*p* = 0.042). However, there were no statistically significant associations between additive intake and disease activity markers such as CRP and hemoglobin. A weak positive correlation was observed between CRP and the Dietary Inflammatory Index (DII) in some regions (r = 0.244, *p* < 0.001), which itself was weakly correlated with total additive intake (r = 0.145, *p* = 0.015).

Finally, Uzunismail et al. conducted a 3-phase provocation trial in adults with CD: a 10-day elimination of IgG-positive foods and additives, followed by 3 days of IgG-positive foods without additives, then 3 days of those foods with additives [39]. Results showed significant increase in CDAI (99.75 ± 46.1, *p* = 0.012), FC (*p* = 0.028), and HBI (*p* = 0.027) during provocation compared to baseline. Together, these findings provide an overview of different contexts that could impact symptoms and disease activity.

## 4. Discussion

The present scoping review identified a range of studies that highlight how the effects of UPF and food additives on IBD activity can vary significantly depending on several factors, such as intake assessment method, UPF classification system, and overall study design. While previous reviews have compiled evidence on how these foods and substances influence IBD development [3,41], this is, to our knowledge, the first review to examine their impact on IBD activity.

### 4.1. Effects of UPF on IBD

Studies on associations between UPF intake and IBD activity demonstrate mixed results. Both studies by Sarbagili-Shabat et al. used the HBI to assess the impact of UPF on disease activity in CD. Their findings show a higher probability of relapse [29] and increased disease activity [28] with higher UPF intake, suggesting an association even outside of long-term follow-ups. These findings build upon previous prospective cohorts linking UPF intake with increased incidence and risk of IBD [42], particularly CD [4,5]. For instance, Narula et al.’s long-term prospective cohort from around the world showed an increased risk of IBD development with high UPF intake [8]. A subsequent systematic review and meta-analysis found that high Nova 4 intake and low Nova 1 intake were associated with increased risk of CD, but not UC [41].

In contrast, Vagianos et al. found more episodes of active disease and inflammation in UC adults with high UPF intake, and not in those with CD [6]. The mixed effects in disease sub-types may be due to variability in outcome measures. For example, while Sarbagili-Shabat et al. used disease-specific tools (HBI for CD and SCCAI for UC) [27], Vagianos et al. used the IBD symptom inventory (IBDSI) for measuring disease activity. IBDSI captures a more comprehensive picture of either subtype as it covers a wider range of symptoms and patient experiences [42]. Nevertheless, all tools have been validated, and while subtype variability must be explored, an association between UPF and IBD activity cannot be discarded.

Koppelman’s prospective cohort and Nitescu’s non-randomized trial showed no associations between UPF intake and disease activity [25,26]. Similarly, Pueschel et al. reported that FC and CRP were not associated with increased HPF intake [27]. However, using an FC and CRP as disease activity measures without a clinical assessment tool such as the HBI or the SCCAI may not capture the entirety of the effects of UPF on IBD [25].

In contrast, Stevens et al.’s unexpected findings showed that red meat, legumes, and vegetables are stronger predictors of IBD flares than UPF [30]. While the literature has shown protective [43] or null [44] effects of vegetables on IBD, dietary fiber is generally suggested to provide protective effects in inflammatory conditions such as IBD, while no strictures are present [45]. Despite unexpected results, Stevens’ novel methodology verifies the notion that food categorization affects the predictive values of foods on IBD flares [30]. This was achieved by using the Sparse Grouped Least Absolute Shrinkage and Selection Operator (SGL) Cox model, where foods are grouped and studied in their ability to predict a specific outcome. Their findings are consistent with previous investigations demonstrating that using different food classification systems influences the association between UPF intake and biochemical markers [46].

Overall, these findings reflect heterogeneity in both methodology and results. Additionally, factors such as socioeconomic status, exposure to drugs, genetic predisposition, and immune response were not accounted for in these investigations, yet they may have a key role in the exacerbation of IBD [47,48].

### 4.2. Role of Food Processing Classification Systems

In the current review, 5 out of 7 studies used the Nova food classification [6,25,28,29,30]. While widely used, Nova has been increasingly criticized for undermining the nutritional value of manufactured food products [49,50]. Nova’s group 4 describes highly palatable products with little to no whole foods that are contained in attractive packaging [4]. However, the focus on formulation disregards the degree of processing and the overall nutritional quality of foods, suggesting misclassification [10,51]. This is important as some UPF groups, such as ultra-processed breads, yogurts, or plant-based foods, have been associated with no risk or decreased risk of cancer and cardiovascular disease and are typically considered as nutritionally appropriate for a healthy diet [52,53].

To avoid the difficulties with Nova, Pueschel et al. used the sQ-HPF to assess HPF intake [27], which incorporates classification criteria from Nova, IARC, IFIC, and UNC systems [54]. Although not a classification system, the sQ-HPF provides an integrative approach to evaluate UPF, as UPF intake is classified as high when it meets the criteria of at least 2 of the 4 systems [54]. Finally, Nitescu et al. developed an exclusion diet based on principles from other anti-inflammatory dietary patterns and their observations on problematic foods in IBD [26]. They provided participants with a list of UPF to avoid, including: all processed meats, store-bought ice creams, condiments, snack foods, flavored yogurt, and fast food. Moreover, the authors highlight that UPF are high in fat, emulsifiers, and often include fried foods. Despite certain similarities with other systems, the arbitrary designation of foods as UPF could signify a source of bias, and leads to further difficulties in comparing results with studies using established systems.

The heterogeneity in classification systems complicates efforts to draw robust conclusions about associations between UPF intake and IBD activity [3]. While recognizing these limitations, it is necessary to explore novel classification systems that also factor in the nutrient profile to better categorize the health matrix of food. The WISEcode-UPF™ system has been mentioned in recent conference abstracts, briefly describing how the classification integrates the number of processed ingredients, level of processing, percentage of calories from added sugars, and ingredients with known concerns [55]. While introducing a new food category designated as “super-ultra” processed, preliminary results suggest a more even distribution of assessed foods across the newly defined 5 categories compared to Nova [55,56].

### 4.3. Effects of Food Additives on IBD

The studies examining food additives were highly heterogeneous. Bhattacharyya et al. conducted a double-blind RCT assessing carrageenan elimination in UC [31]. While the study had a strong design in terms of blinding and control, it had a very small sample size (*n* = 12), reducing the statistical power and thus restricting its generalizability. In contrast, Fitzpatrick et al. studied emulsifiers in a double-blind randomized feeding design, where participants were provided with all their meals under a controlled setting [32]. This approach helped reduce variability in intake and improved consistency, but again, the sample size (*n* = 15) and short intervention duration restrict the strength of the conclusion that can be drawn.

The DELECTABLE program provides compelling evidence that structured dietary interventions are both feasible and effective in IBD care [38]. Patients with CD and UC not only reported high satisfaction and adherence, but also demonstrated significant reductions in disease activity scores, inflammatory markers, and improvements in quality of life over 12-week period. At the same time, cautious interpretation is warranted. The study is open-label and non-randomized, limiting comparability across diet arms and risking confounding by patient preference and concurrent medical therapy changes. Moreover, long-term efficacy and sustainability remain open questions, as the 12-week time frame does not elucidate maintenance of remission. Nevertheless, given the difficulties in isolating the effects of food additives, whole-diet interventions may propose a more realistic approach to investigating diet therapy for IBD patients compared to exclusion diets.

Emulsifiers have emerged as a key category of food additives hypothesized to influence disease activity in IBD [57], but study results remain inconsistent. In a tightly controlled feeding trial [32] comparing diets with high and low emulsifier content in CD patients, both groups showed improvements in clinical symptoms and intestinal sonographic measures. Additionally, there were no statistically significant differences between the groups in clinical and biochemical parameters. This suggests that within the context of a healthy diet, emulsifier content alone may not have a significant impact on disease activity within a short timeframe. Although the study lacked dietary control, Sandall et al. reported symptomatic and biomarker improvements following emulsifier exclusion in UC patients [34]. Similarly, Marsh et al. incorporated emulsifier restriction within their dietary intervention (IBD-MAID) and observed significant decreases in FC, yet no differences in CDAI and SCCAI [37]. These conflicting findings may stem from differences in disease populations, blinding and control, intervention duration, as well as inconsistency in both the modification of emulsifiers and dietary intake assessment. Overall, existing trials do not provide definitive evidence that exclusion of emulsifiers changes disease course or affects its activity.

Two placebo-controlled randomized trials have explored the impact of carrageenan in individuals with ulcerative colitis, yet reached different conclusions despite similar designs [31,33]. Bhattacharyya et al. revealed that the introduction of carrageenan in the capsule form triggered clinical relapse and increased FC in patients with UC, suggesting a pro-inflammatory role for the additive [31]. While Laatikainen et al. found no statistically significant differences in clinical or biochemical markers when patients consumed carrageenan-containing diets versus carrageenan-free diets [33]. This discrepancy may reflect key differences in carrageenan delivery and dosing; Bhattacharyya used isolated capsules (100 mg), although less than the daily average intake, while Laatikainen used food-grade carrageenan at dietary levels (2000 mg) incorporated into regular foods. Notably, the lack of observable impact at higher intake levels in Laatikainen’s study raises questions about whether carrageenan exposure would cause significant inflammation. Moreover, the duration of exposure differed substantially, with the latter’s intervention being much shorter, possibly limiting the ability to capture cumulative inflammatory effects. These findings suggest that the inflammatory role of carrageenan may depend heavily on dose, formulation, and duration of exposure.

Similarly, Zorich et al. suggested that olestra, a fat substitute, had no negative effects on IBD [40]. However, funding for this study was provided by Procter & Gamble, a company responsible for the development of olestra and its later introduction to the food industry, which suggests possible bias. Notable, since then, olestra has been prohibited in Canada and the European Union.

Several studies reported changes in inflammatory biomarkers such as FC or CRP following dietary interventions [36,37,39], suggesting potential mucosal susceptibility to diet. However, translating these findings into the context of IBD remains challenging due to the differences in additive formulation, host susceptibility, and complexity of whole-diet exposures. Furthermore, while reductions in FC suggest some degree of mucosal healing, these changes are not always mirrored by significant clinical improvements. This highlights the need for future research aimed at discerning the contribution of food additives using standardized exposure assessment methods.

### 4.4. Strengths and Limitations

The available evidence assessing the impact of UPF and food additives on IBD outcomes is still emerging and presents several limitations. One major challenge is the variability in how intake of UPFs and food additives is assessed, which was mainly performed using unvalidated tools that have a high risk of recall bias, making it difficult to ensure accuracy across studies [58]. Additionally, the lack of uniformity in disease activity measurements reflects another important limitation in the available evidence. While this variability poses difficulties in comparing the evidence, all the measures used across the included studies are validated in their respective population, which strengthens the reliability of findings.

Additionally, many of the included studies on food additives were conducted in relatively small sample sizes, and some had short follow-up periods, which limits statistical power and decreases the generalizability of findings. These limitations are likely attributed to the difficulty recruiting participants willing to participate in interventions, including the intake of controversial substances, such as emulsifiers. However, the included studies cover a wide range of countries, meaning that findings could have validity in different contexts.

A main limitation in the current review is that some retrieved studies were only available as abstracts or registered trials with incomplete results/data, which may have led to underrepresentation of relevant findings. Moreover, a formal quality appraisal was not conducted for the included studies, which presents obstacles in assessing the reliability of the collective findings. Finally, associations between UPF intake and biochemical markers vary by the classification system used; standardizing definitions or performing sensitivity analyses could improve comparability across studies [46].

A notable strength of this scoping review is the focus on the impact of UPF and food additives specifically on disease activity and outcomes in patients with established IBD. While much of the existing evidence focuses on the role of dietary factors in the development of IBD, fewer studies have examined how dietary exposures influence the disease course and its outcomes. This review provides a comprehensive summary of the current literature on this topic and highlights the gaps to guide further research.

Mechanistically, many of the implicated additives have been shown to disrupt the intestinal epithelium and alter the gut microbiome [10,58]. However, our review did not systematically assess microbiome-focused studies, which represents a limitation given their potential relevance to understanding how diet affects the GI mucosa and clinical outcomes.

### 4.5. Implications

Given the increased UPF intake, the current review is relevant to bring attention to the potential effects of these foods on inflammatory conditions such as IBD. Additionally, the discrepancies between studies highlight the need for standardized food classification systems to focus on the health impacts of foods and substances. Finally, the current review shows the need to investigate the effects of UPF and food additives under more rigorous study designs, which will help generate high-quality evidence capable of informing future clinical practice guidelines.

### 4.6. Research Gaps and Future Directions

Many knowledge gaps have been identified with this scoping review. First, it remains unknown whether there is a dose–response relationship between UPF intake and IBD activity. Secondly, it would be beneficial to understand whether covariates such as other comorbidities exacerbate the effects of these foods and substances on IBD activity. Thus, further investigations with different study designs would capture further details of the associations. Additionally, longer follow-up periods in both UPF and food additive intake would allow for a better understanding of the outcomes. Finally, the standardization of UPF classification would greatly facilitate further reviews investigating similar questions and aid in the understanding of the role of UPF in other disease states.

## 5. Conclusions

The growing body of evidence shows considerable variability in the effects of UPF on IBD activity and symptoms, with studies reporting positive, negative, and null findings. These inconsistencies may reflect the influence of external factors and study methodology. Similarly, the evidence on the effects of food additives remains variable, and studies on additives of interest, such as emulsifiers, show conflicting results. Considering IBD is a condition where phenotype, treatment, and external factors impact disease outcomes, future research must account for these factors to better elucidate the role of these foods and substances in disease activity.

## Figures and Tables

**Table 1:** Inclusion and exclusion criteria.

Inclusion Criteria	Exclusion Criteria
Studies involving adults (≥18 years) diagnosed with IBD Studies examining the intake of UPF and/or food additives according to any classification system Studies that assess disease outcomes including disease activity, flare frequency, or IBD-related GI symptoms Observational studies, including cohort, case–control, and cross-sectional designs Articles published in English, French, and Spanish	Non-human or in vitro studies Studies not reporting outcomes related to disease activity or symptomatology Studies not distinguishing IBD from other conditions (e.g., IBS, colorectal cancer) unless IBD-specific results are provided Review articles (they will be used for a manual reference search) Studies focused solely on pediatric populations or non-IBD GI diseases Studies assessing microbiome-related data

IBD; inflammatory bowel disease, UPF; Ultra-processed foods, GI; gastrointestinal, IBS; inflammatory bowel syndrome.

**Table 2:** Description of UPF according to different classification systems.

Classification Systems	UPF Definition
Nova	Group 4: Industrial formulations with substances not commonly used at home and food additives to improve sensory qualities. Processes include extrusion, moulding, or hydrogenation [11].
IARC	Group 3: Industrially prepared foods requiring no or minimal domestic preparation besides heating or cooking [12].
IFIC	Group 4: Packaged foods and store-prepared mixtures high in total and added sugars and low content of dietary fiber. Group 5: Foods packaged to facilitate preparation. For example, frozen meals, entrées, and prepared deli foods [13].
UNC	Group 4: Industrially formulated foods and beverages with multiple ingredients and that become unrecognizable as their original form due to extensive processing [14].

UPF; ultra-processed food, IARC; International Agency for Research on Cancer, IFIC; International Food Information Council, UNC; University of North Carolina.

**Table 3:** Summary of the included studies on UPF.

Author, Year, and Country	Sample and Population	Study Design	Study Funding	UPF Classification and Intake Assessment Method	Outcome Measures	Impact on Disease Activity or Symptoms
Koppelman et al. [25] 2025 The Netherlands	*n* = 191 Adults with CD (53.9%) and UC (46.1%).	1-year prospective cohort. Exposure: UPF intake.	Alphasigma USA.	Nova classification. GINQ-FFQ.	1 outcome: Disease activity (CRP and FC). 2 outcome: FrQoL.	No differences in UPF intake between patients in remission and active disease, or between baseline and the end of the study.
Nitescu et al. [26] 2023 Romania	*n* = 139 Adults with UC (44.6%) and CD (55.4%) in remission.	6-month non-randomized trial. Intervention: Exclusion diet. Control: Regular diet.	No external funding reported.	Classification created by authors. Questionnaire assessing dietary habits.	1 outcome: Disease activity (Mayo score and CDAI). 2 outcomes: CRP, ESR, and FC.	100% of the exclusion group and 95.7% of the control group maintained clinical remission after 6 months. FC ↑ in both groups, but between-group differences are not significant.
Pueschel et al. [27] 2025 Germany	*n* = 233 Adults with CD (60.5%) and UC (39.5%).	Cross-sectional subanalysis of a prospective cohort.	Hannover Medical School.	sQ-HPF classification. FFQ and sQ-HPF.	1 outcome: Disease activity (HBI and PMS). 2 outcomes: SHS, Fr-QoL-29, FC and CRP.	Significantly higher HPF% intake in IBD subjects compared to the control cohort (*p* < 0.001). No significant correlations between HPF% and FC, CRP in entity- and sex-stratified groups.
Sarbagili-Shabat et al. [28] 2024 Israel	*n* = 242 Adults with CD (62.8%) and UC (37.2%).	Cross-sectional study.	No external funding reported.	Nova classification. PFQ and FFQ.	1 outcome: Active disease (HBI and SCCAI).	High UPF intake was associated with active disease (OR = 3.82, 95% CI: 1.49–9.8). High intake of unprocessed, minimally processed, and processed foods was negatively associated with active disease.
Sarbagili-Shabat et al. [29] 2025 Israel	*n* = 111 Adults with CD on clinical remission.	1-year prospective cohort. Exposure: UPF consumption.	No external funding reported.	Nova classification. PFQ and FFQ.	1 outcome: Clinical relapse (HBI, disease activity requiring change in medication, hospitalization, or IBD-related surgery). 2 outcome: FC.	Relapse at 1 year ↑ in high UPF intake (>3.6 servings/day) vs. low UPF intake (*p* = 0.032). High UPF ↑ risk of clinical relapse (HR = 3.86; 95% CI 1.30–11.47).
Stevens et al. [30] 2025 The Netherlands	*n* = 724 Adults with CD and UC.	2-year prospective cohort. Exposure: Dietary pattern.	European Union 7th Framework Program.	Nova classification. FFQ.	1 outcome: Flare development (presence of active disease via endoscopy or radiography, FC, HBI, SCCAI, CRP).	When categorizing foods into UPF vs. non-UPF, all non-UPF ↑ impact on flare prediction compared to UPF.
Vagianos et al. [6] 2024 Canada	*n* = 135 Adults with CD (65.2%) and UC (34.8%).	1-year prospective cohort. Exposure: UPF consumption.	Canadian Institutes of Health Research.	Nova classification. Harvard FFQ.	1 outcomes: Active disease (IBDSI), and active inflammation (FC).	Among patients with UC, higher UPF intake ↑ episodes of active disease and inflammation. No significant association found in CD patients.

Legend: Crohn’s disease (CD), ultra-processed food (UPF), processed food questionnaire (PFQ), food frequency questionnaire (FFQ), Harvey Bradshaw index (HBI), inflammatory bowel disease (IBD), fecal calprotectin (FC), hazard ratio (HR), confidence interval (CI), IBD symptom inventory (IBDSI), ulcerative colitis (UC), Crohn’s disease activity index (CDAI), C-reactive protein (CRP), erythrocyte sedimentation rate (ESR), Groningen IBD nutritional questionnaire (GINQ-FFQ), food-related quality of life (FrQoL), simple clinical colitis activity index (SCCAI), odds ratio (OR), screening questionnaire of highly processed foods (sQ-HPF), partial Mayo score (PMS), short health scale (SHS), highly processed foods (HPF), increase (↑).

**Table 4:** Summary of the included studies on food additives.

Author, Year, and Country	Sample and Population	Study Design	Study Funding	Food Additive Name or Type and Intake Assessment Method	Outcome Measures	Impact on Disease Activity or Symptoms
Bhattacharyya et al. [31] 2017 USA	*n* = 12 Adults with UC in clinical remission ≥ 1 month.	1-year randomized double-blind, placebo-controlled trial. Intervention: Carrageenan capsules. Control: Placebo.	No external funding reported.	Carrageenan. Assessment: 24 h dietary recalls every 2 weeks.	1 outcome: Occurrence of relapse (increase of ≥ 2 points on SCCAI) in association with increase in treatment. 2 outcomes: QOL (SIBDQ), inflammation (FC, IL-6).	The carrageenan group ↑ relapse risk vs. Placebo group (*p* = 0.046). No relapses in the placebo group. FC and IL-6 ↑ in the carrageenan group (*p* = 0.06, *p* = 0.02, respectively).
Fitzpatrick et al. [32] 2025 Australia	*n* = 24 Adults with active CD, on ≥ 2 months stable therapy.	4 weeks randomized double-blinded feeding study. Intervention: HED or LED.	Crohn’s and Colitis Australia PhD Scholarship, NHMRC, Crohn’s and Colitis Foundation Litwin Pioneers Program.	Emulsifiers and thickeners. Assessment: food diaries.	Exploratory outcomes: Disease activity (HBI), GIUS, FC, CRP, specific symptoms, and QOL (IBDQ).	9/12 on HED and 7/12 on LED were in clinical remission at the end of intervention. No significant difference in sonographic response between groups: 7/12 in HED and 4/12 in LED. FC remained unchanged after both interventions.
Laatikainen et al. [33] 2023 Finland	*n* = 7 Adults with quiescent UC.	28-day randomized cross-over trial. Intervention: 7 days of carrageenan, 7 days of placebo, and 14 days of wash-out.	Juhani Aho Medical Research Foundation, Folkhälsan Research Center, Wilhelm and Else Stockmann Foundation, and Novo Nordisk Foundation.	Carrageenan. Assessment: 3-day food diaries.	1 outcome: Disease activity (SCCAI). 2 outcomes: GI symptoms (VAS), Hs-CRP, FC.	No statistically significant difference in SCCAI between carrageenan and placebo periods, or when each period was compared to baseline. No significant differences between treatments in any biochemical parameters.
Sandall. [34] 2021 London	*n* = 10 Adults with active CD.	8-week RCT. Intervention: LED. Control: emulsifier-containing diet.	Helmsley Charitable Trust	Emulsifiers. Assessment: 7-day food diaries.	1 outcome: CDAI reduction. 2 outcomes: FC, IBD-Control questionnaire, health-related QOL (IBDQ), food-related QOL.	4 participants achieved CDAI reduction, but results were not statistically significant when comparing CDAI from baseline to the end of the trial. Patient-perceived disease control ↑ at the end of the trial (*p* = 0.015).
Lomer et al. [35] 2004 England	*n* = 83 Adults with active CD.	16-week randomized placebo-controlled trial. Treatments: LCLM, LCNM, NCLM, NCNM.	NHS Executive, the PPP Foundation, and the DH NHS R&D Programme.	TiO_2_ (food colorant) and Psil (anti-caking agent). Assessment: dietary recall.	1 outcome: Remission or clinical response (CDAI). 2 outcomes: IBDQ, ESR, CRP, FC, intestinal permeability, and Van Hees Index.	No significant differences in remission rates or clinical response between low and normal microparticle groups. No difference between the low and normal microparticle groups for any secondary outcomes.
Day et al. [36] 2022 Australia	*n* = 28 Adults with mild to moderately active UC.	8-week prospective feasibility study. Intervention: self-application of 4-SURE diet.	No external funding reported.	Restriction of sulfur-containing amino acids, sulfite/sulfate, nitrite/nitrate, and carrageenan additives. Assessment: self-reported intake.	1 outcome: Diet tolerability. 2 outcomes: Self-reported adherence, partial Mayo clinical response, endoscopic and fecal fermentation markers, food-related QOL.	Clinical response in 46% (13/28) and endoscopic improvement in 36% (10/28). Unchanged CRP, median FC ↓ by 131 µg/g (*p* = 0.02). Fecal concentration of SCFAs ↑ by 17%.
Marsh et al. [37] 2024 Australia	*n* = 58 Adults with UC (75%) or CD (25%).	16-week RCT. Intervention: IBD MAID. Control: General healthy eating education.	Australian Government Research Training Program Scholarship	Non-nutritive sweeteners, nitrites/nitrates, maltodextrin, P80, CMC, carrageenan gum, and ‘other’ emulsifiers. Assessment: 3-day food diaries and a food additive score.	1 outcomes: Disease activity (SCCAI and CDAI). 2 outcomes: SIBDQ, PRO2 questionnaire, FC, CRP.	CDAI ↓ from baseline to week 8 in both groups, but the difference between groups was not significant. No difference between groups in SCCAI. FC ↓ in the intervention group at the end (*p* = 0.002).
Trakman et al. [38] 2025 Australia.	*n* = 64 Adults with CD (58%), UC (39%), or microscopic colitis (3%).	12-week interventional prospective study. Interventions: CDED, WFD, and UCD.	Australasian Gastrointestinal Research Foundation, and a charitable donation from the Yencken family.	CDED: exclusion of inflammatory foods. WFD: exclusion of UPF and food additives. UCD: restriction of sulfur-containing components. Assessment: FFQ and 24 h recalls.	1 outcomes: diet satisfaction (DSAT-28), adherence (self-reported and dietitian-rated), food additive intake. 2 outcomes: QOL (IBDQ-9), CDAI, partial Mayo score, CRP, FC.	CDAI ↓ on CDED *(p* = 0.023) and WFD (*p* = 0.038). CRP ↓ on CDED (*p* = 0.043). Partial Mayo score improved on WFD (*p* = 0.02). No significant changes in the UCD.
Trakman et al. [9] 2022 Australia and China.	*n* = 274 Adults with CD from 3 cohorts (Australia, Hong Kong, and China).	Cross-sectional case–control study.	Helmsley Charitable Trust.	Intake of processed foods and food additives. Assessment: FFQ and 3-day food diaries.	1 outcomes: Disease activity (CDAI), CRP, Hgb, DII, and additive intake in relation to current CRP and Hgb ranges.	Weak positive correlation between DII and CRP. No significant group-level differences in nutrient intake by inflammation markers.
Uzunismail et al. [39] 2011 Turkey.	*n* = 8 Adults with CD in remission, previously on IgG elimination diet for 6–30 months.	Provocation trial: 10-day elimination of IgG-positive additives, 3 days of provocation with pure IgG-positive foods, and 3 days of off-the-shelf forms of IgG foods.	No external funding reported.	Intake of foods and additives chosen based on elevated IgG antibody levels. Assessment: food diaries.	1 outcome: Disease activity (CDAI and HBI). 2 outcomes: FC, CRP, ESR.	CDAI score and HBI score ↑ during provocation (*p* = 0.012, *p* = 0.027, respectively). WBC and CRP levels ↑ after provocation (*p* = 0.036 and *p* = 0.025, respectively). FC ↑ during provocation after excluding 1 patient due to noncompliance with diet (*p* = 0.018).
Zorich et al. [40] 1997 USA.	*n* = 83 Adults with CD (51%) and UC (41%) in remission.	4-week RCT. Intervention: 20 g olestra. Control: 20 g conventional vegetable triglycerides.	Procter & Gamble.	Olestra. Assessment: food diaries.	1 outcome: CDAI, flexible sigmoidoscopy. 2 outcome: Bowel permeability.	No significant difference between both groups in the percentage of patients who relapsed, or the percentage of patients who experienced worsening of their symptoms.

Legend: Ulcerative colitis (UC), simple clinical colitis activity index (SCCAI), quality of life (QOL), short inflammatory bowel disease questionnaire (SIBDQ), fecal calprotectin (FC), interleukin-6 (IL-6), Crohn’s disease (CD), high emulsifier diet (HED), low emulsifier diet (LED), National Health & Medical Research Council Investigator Grant (NHMRC), Harvey-Bradshaw index (HBI), gastrointestinal ultrasound (GIUS), C-reactive protein (CRP), inflammatory bowel disease questionnaire (IBDQ), gastrointestinal (GI), virtual analogue scale (VAS), high-sensitivity C-reactive protein (Hs-CRP), randomized controlled trial (RCT), Crohn’s disease activity index (CDAI), low calcium low microparticles (LCLM), low calcium normal microparticles (LCNM), normal calcium low microparticles (NCLM), normal calcium normal microparticles (NCNM), titanium dioxide (TiO_2_), particulate silicates (Psil), erythrocyte sedimentation rate (ESR), 4 strategies for sulfide reduction (4-SURE), short-chain fatty acids (SCFAs), MAID (modified anti-inflammatory diet), polysorbate 80 (P80), carboxymethylcellulose (CMC), patient reported outcomes-2 (PRO2), Crohn’s disease exclusion diet (CDED), Wholefood diet (WFD), Ulcerative colitis diet (UCD), food frequency questionnaire (FFQ), diet satisfaction questionnaire (DSAT-28), hemoglobin (Hgb), dietary inflammatory index (DII), immunoglobulin G (IgG), white blood cell count (WBC), increase (↑), decrease (↓).

## Data Availability

No new data were created or analyzed in this study. Data sharing is not applicable to this article.

## Supplementary Materials

The following supporting information can be downloaded at: https://www.mdpi.com/article/10.3390/jcm14217798/s1, Supplement S1: Preferred Reporting Items for Systematic reviews and Meta-Analyses extension for Scoping Reviews (PRISMA-ScR) Checklist. Reference [59] are cited in the supplementary materials.

## Abbreviations

The following abbreviations are used in this manuscript:

IBD	Inflammatory bowel disease
CD	Crohn’s disease
UC	Ulcerative colitis
UPF	Ultra-processed foods
GI	Gastrointestinal
FC	Fecal calprotectin
CRP	C-reactive protein
HBI	Harvey-Bradshaw index
SCCAI	Simple clinical colitis activity index
CDAI	Crohn’s disease activity index

## Appendix A

**Table A1:** Search strategy used on Medline, including the key terms and the queries.

Date and Database	Line	Key Terms, Queries, and Number of Results
25 June 2025 Medline	1	exp Inflammatory Bowel Diseases/(106,365)
	2	(inflammat* adj3 bowel* adj3 disease*).tw,kf. (76,262)
	3	1 or 2 (137,017)
	4	Colitis, Ulcerative/(44,756)
	5	((ulcerat* or gravis) adj3 colitis).tw,kf. (57,727)
	6	(idiopathic adj2 proctocolitis).tw,kf. (37)
	7	4 or 5 or 6 (66,570)
	8	Crohn Disease/(47,538)
	9	(crohn* adj3 (disease or enteritis)).tw,kf. (61,763)
	10	((granulomatous or regional) adj3 (enteritis or colitis)).tw,kf. (1866)
	11	(ileitis adj3 (terminal or regional)).tw,kf. (1373)
	12	8 or 9 or 10 or 11 (72,183)
	13	Food, Processed/(624)
	14	((ultra-process* or highly-process* or mass-produced or transformed or commercially-prepared) adj3 (food* or packaged food*)).tw,kf. (2799)
	15	13 or 14 (2896)
	16	exp Food Additives/(346,079)
	17	(food* adj2 (additive* or colo?ring*)).tw,kf. (10,285)
	18	(preservative* or emulsifier* or thickener* or stabilizer* or gelling or sweetener*).tw,kf. (54,215)
	19	((emulsify* or thicken* or stabiliz* or gelling) adj2 agent*).tw,kf. (7107)
	20	((colo?r or flavo?r or sweetener*) adj2 (enhancer* or artificial or non-nutritive)).tw,kf. (2643)
	21	16 or 17 or 18 or 19 or 20 (403,506)
	22	3 or 7 or 12 (154,009)
	23	15 or 21 (406,164)
	24	22 and 23 (1037)

Note: * is used as a truncation symbol.

## References

[1] Kaplan G.G., Ng S.C. (2017). Understanding and preventing the global increase of inflammatory bowel disease. Gastroenterology.

[2] Lin D., Jin Y., Shao X., Xu Y., Ma G., Jiang Y., Xu Y., Jiang Y., Hu D. (2024). Global, Regional, and National Burden of Inflammatory Bowel Disease, 1990–2021: Insights from the Global Burden of Disease 2021. Int. J. Color. Dis..

[3] Babaei A., Pourmotabbed A., Talebi S., Mehrabani S., Bagheri R., Ghoreishy S.M., Amirian P., Zarpoosh M., Mohammadi H., Kermani M.A.H. (2024). The association of ultra-processed food consumption with adult inflammatory bowel disease risk: A systematic review and dose-response meta-analysis of 4035694 participants. Nutr. Rev..

[4] Chen J., Wellens J., Kalla R., Fu T., Deng M., Zhang H., Yuan S., Wang X., Theodoratou E., Li X. (2023). Intake of ultra-processed foods is associated with an increased risk of Crohn’s disease: A cross-sectional and prospective analysis of 187154 participants in the UK Biobank. J. Crohns Colitis..

[5] Lo C.-H., Khandpur N., Rossato S.L., Lochhead P., Lopes E.W., Burke K.E., Richter J.M., Song M., Korat A.V.A., Sun Q. (2022). Ultra-processed foods and risk of Crohn’s disease and ulcerative colitis: A prospective cohort study. Clin. Gastroenterol. Hepatol..

[6] Vagianos K., Dolovich C., Witges K., Graff L.A., Bernstein C.N. (2024). Ultra-processed food, disease activity, and inflammation in ulcerative colitis: The Manitoba Living with IBD Study. Am. J. Gastroenterol..

[7] Jarmakiewicz-Czaja S., Piątek D., Filip R. (2022). The impact of selected food additives on the gastrointestinal tract in the example of nonspecific inflammatory bowel diseases. Arch. Med. Sci..

[8] Narula N., Wong E.C.L., Dehghan M., Mente A., Rangarajan S., Lanas F., Lopez-Jaramillo P., Rohatgi P., Lakshmi P.V.M., Varma R.P. (2021). Association of ultra-processed food intake with risk of inflammatory bowel disease: Prospective cohort study. BMJ.

[9] Trakman G.L., Lin W.Y.Y., Hamilton A.L., Wilson-O’brien A.L., Stanley A., Ching J.Y., Yu J., Mak J.W.Y., Sun Y., Niu J. (2022). Processed food as a risk factor for the development and perpetuation of Crohn’s disease—The ENIGMA study. Nutrients.

[10] Spiller A.L., Costa B.G.d., Yoshihara R.N.Y., Nogueira E.J.Z., Castelhano N.S., Santos A., Brusco De Freitas M., Magro D.O., Yukie Sassaki L. (2025). Ultra-Processed Foods, Gut Microbiota, and Inflammatory Bowel Disease: A Critical Review of Emerging Evidence. Nutrients.

[11] de Araújo T.P., de Moraes M.M., Afonso C., Santos C., Rodrigues S.S.P. (2022). Food processing: Comparison of different food classification systems. Nutrients.

[12] Monteiro C.A., Cannon G., Levy R.B., Moubarac J.C., Jaime P., Martins A.P., Canella D., Louzada M.L., Parra D. (2016). NOVA. The star shines bright. Food classification. Public health. World Nutr..

[13] Slimani N., Deharveng G., Southgate D.A.T., Biessy C., Chajès V., Van Bakel M.M.E., Boutron-Ruault M.C., McTaggart A., Grioni S., Verkaik-Kloosterman J. (2009). Contribution of highly industrially processed foods to the nutrient intakes and patterns of middle-aged populations in the European Prospective Investigation into Cancer and Nutrition study. Eur. J. Clin. Nutr..

[14] Eicher-Miller H.A., Fulgoni V.L., Keast D.R. (2012). Contributions of processed foods to dietary intake in the US from 2003–2008: A report of the Food and Nutrition Science Solutions Joint Task Force of the Academy of Nutrition and Dietetics, American Society for Nutrition, Institute of Food Technologists, and International Food Information Council. J. Nutr..

[15] Poti J.M., Mendez M.A., Ng S.W., Popkin B.M. (2015). Is the degree of food processing and convenience linked with the nutritional quality of foods purchased by US households?. Am. J. Clin. Nutr..

[16] Monteiro C.A., Cannon G., Levy R.B., Moubarac J.-C., Louzada M.L., Rauber F., Khandpur N., Cediel G., Neri D., Martinez-Steele E. (2019). Ultra-Processed Foods: What They Are and How to Identify Them. Public Health Nutr..

[17] Sadler C.R., Grassby T., Hart K., Raats M., Sokolović M., Timotijevic L. (2021). Processed Food Classification: Conceptualisation and Challenges. Trends Food Sci. Technol..

[18] Bleiweiss-Sande R., Chui K., Evans E.W., Goldberg J., Amin S., Sacheck J. (2019). Robustness of Food Processing Classification Systems. Nutrients.

[19] Isaksen I.M., Dankel S.N. (2023). Ultra-processed food consumption and cancer risk: A systematic review and meta-analysis. Clin. Nutr..

[20] Dai S., Wellens J., Yang N., Li D., Wang J., Wang L., Yuan S., He Y., Song P., Munger R. (2024). Ultra-processed foods and human health: An umbrella review and updated meta-analyses of observational evidence. Clin. Nutr..

[21] Ng S.C., Shi H.Y., Hamidi N., Underwood F.E., Tang W., Benchimol E.I., Panaccione R., Ghosh S., Wu J.C.Y., Chan F.K.L. (2017). Worldwide incidence and prevalence of inflammatory bowel disease in the 21st century: A systematic review of population-based studies. Lancet.

[22] Baker P., Machado P., Santos T., Sievert K., Backholer K., Hadjikakou M., Russell C., Huse O., Bell C., Scrinis G. (2020). Ultra-processed foods and the nutrition transition: Global, regional and national trends, food systems transformations and political economy drivers. Obes. Rev..

[23] Page M.J., McKenzie J.E., Bossuyt P.M., Boutron I., Hoffmann T.C., Mulrow C.D., Shamseer L., Tetzlaff J.M., Akl E.A., Brennan S.E. (2021). The PRISMA 2020 statement: An updated guideline for reporting systematic reviews. BMJ.

[24] Ouzzani M., Hammady H., Fedorowicz Z., Elmagarmid A. (2016). Rayyan—A web and mobile app for systematic reviews. Syst. Rev..

[25] Koppelman L.J.M., Stevens C.L., Barth I., Jacobs R.J., Dijkstra G., van der Meulen-de Jong A.E., Campmans-Kuijpers M.J.E. (2025). Can diet quality be associated with disease activity in a prospective Dutch inflammatory bowel disease cohort?. Nutrients.

[26] Nitescu M., Istratescu D., Preda C.M., Manuc T.E., Louis E., Manuc M., Stroie T., Catrinoiu M., Tieranu C.G., Badea L.E. (2023). Role of an exclusion diet (reduced disaccharides, saturated fats, emulsifiers, red and ultraprocessed meats) in maintaining the remission of chronic inflammatory bowel diseases in adults. Medicina.

[27] Pueschel L., Nothacker S., Kuhn L., Wedemeyer H., Lenzen H., Wiestler M. (2025). Assessing highly processed food consumption in patients with inflammatory bowel disease: Application of the German Screening Questionnaire (sQ-HPF). J. Clin. Med..

[28] Sarbagili-Shabat C., Zelber-Sagi S., Isakov N.F., Hirsch A., Ron Y., Grinshpan L.S., Anbar R., Bromberg A., Thurm T., Maharshak N. (2024). Ultra-processed foods consumption is positively associated with the clinical activity of inflammatory bowel diseases: A cross-sectional single-center study. Inflamm. Intest. Dis..

[29] Sarbagili-Shabat C., Zelber-Sagi S., Fliss Isakov N., Hirsch A., Ron Y., Sol Grinshpan L., Cohen A., Leibovitzh N., Thurm H., Maharshak N. (2025). High ultra-processed food consumption is associated with clinical exacerbation in patients with Crohn’s disease in remission: A prospective cohort study. Dig. Dis..

[30] Stevens C.L., Adriaans G.M., Spooren C.E., Peters V., Pierik M.J., Weersma R.K., van Dullemen H.M., Festen E.A., Visschedijk M.C., Hendrix E.M. (2025). Exploring diet categorizations and their influence on flare prediction in inflammatory bowel disease, using the Sparse Grouped Least Absolute Shrinkage and Selection Operator method. Clin. Nutr..

[31] Bhattacharyya S., Shumard T., Xie H., Dodda A., Varady K.A., Feferman L., Halline A.G., Goldstein J.L., Hanauer S.B., Tobacman J.K. (2017). A randomized trial of the effects of the no-carrageenan diet on ulcerative colitis disease activity. Nutr. Healthy Aging.

[32] Fitzpatrick J.A., Gibson P.R., Taylor K.M., Anderson E.J., Friedman A.B., Ardalan Z.S., Smith R.L., Halmos E.P. (2025). Clinical trial: The effects of emulsifiers in the food supply on disease activity in Crohn’s disease: An exploratory double-blinded randomised feeding trial. Aliment. Pharmacol. Ther..

[33] Laatikainen R., Lehto M., Mäkelä-Salmi N., Hillilä M., Groop P.H., Salmenkari H. (2023). Randomized controlled pilot study: Effect of carrageenan emulsifier on inflammation and gastrointestinal symptoms in quiescent ulcerative colitis. Food Nutr. Res..

[34] Sandall A. (2022). Food-Additive Emulsifiers and the Low Emulsifier Diet in Crohn’s Disease. Ph.D. Thesis.

[35] Lomer M.C., Grainger S.L., Ede R., Catterall A.P., Greenfield S.M., Cowan R.E., Vicary F.R., Jenkins A.P., Fidler H., Harvey R.S. (2005). Lack of efficacy of a reduced microparticle diet in a multi-centred trial of patients with active Crohn’s disease. Eur. J. Gastroenterol. Hepatol..

[36] Day A.S., Yao C.K., Costello S.P., Ruszkiewicz A., Andrews J.M., Gibson P.R., Bryant R.V. (2022). Therapeutic potential of the 4 strategies to SUlfide-REduction (4-SURE) diet in adults with mild to moderately active ulcerative colitis: An open-label feasibility study. J. Nutr..

[37] Marsh A., Chachay V., Banks M., Okano S., Hartel G., Radford-Smith G. (2024). A pilot randomized controlled trial investigating the effects of an anti-inflammatory dietary pattern on disease activity, symptoms and microbiota profile in adults with inflammatory bowel disease. Eur. J. Clin. Nutr..

[38] Trakman G.L., Russell E.E., Hamilton A.L., Wilson-O’Brien A., Thompson E., Simmance N., Niewiadomski O., Kamm M.A. (2025). Practical application of evidence-based dietary therapy in inflammatory bowel disease: The DELECTABLE program. Nutrients.

[39] Uzunısmaıl H., Cengız M., Uzun H., Ozbakir F., Göksel S., Demırdağ F., Can G., Balci H. (2012). The effects of provocation by foods with raised IgG antibodies and additives on the course of Crohn’s disease: A pilot study. Turk. J. Gastroenterol..

[40] Zorich N.L., Jones M.B., Kesler J.M., Carter S.B., Sutton M.A., Bayless T. (1997). A randomized, double-blind study of the effect of olestra on disease activity in patients with quiescent inflammatory bowel disease. Olestra in IBD Study Group. Am. J. Med..

[41] Narula N., Chang N.H., Mohammad D., Wong E.C.L., Ananthakrishnan A.N., Chan S.S.M., Carbonnel F., Meyer A. (2023). Food processing and risk of inflammatory bowel disease: A systematic review and meta-analysis. Clin. Gastroenterol. Hepatol..

[42] Sexton K.A., Walker J.R., Targownik L.E., Graff L.A., Haviva C., Beatie B.E., Petty S.K., Bernstein M.T., Singh H., Miller N. (2019). The Inflammatory Bowel Disease Symptom Inventory: A patient-report scale for research and clinical application. Inflamm. Bowel Dis..

[43] Milajerdi A., Ebrahimi-Daryani N., Dieleman L.A., Larijani B., Esmaillzadeh A. (2021). Association of dietary fiber, fruit, and vegetable consumption with risk of inflammatory bowel disease: A systematic review and meta-analysis. Adv. Nutr..

[44] Deng M., Dan L., Ye S., Chen X., Fu T., Wang X., Chen J. (2023). Higher dietary fibre intake is associated with lower risk of inflammatory bowel disease: Prospective cohort study. Aliment. Pharmacol. Ther..

[45] Lamb C.A., Kennedy N.A., Raine T., Hendy P.A., Smith P.J., Limdi J.K., Hayee B., Lomer M.C.E., Parkes G.C., Selinger C. (2019). British Society of Gastroenterology consensus guidelines on the management of inflammatory bowel disease in adults. Gut.

[46] Martinez-Perez C., San-Cristobal R., Guallar-Castillon P., Martínez-González M., Salas-Salvadó J., Corella D., Castañer O., Martinez J., Alonso-Gómez Á.M., Wärnberg J. (2021). Use of Different Food Classification Systems to Assess the Association between Ultra-Processed Food Consumption and Cardiometabolic Health in an Elderly Population with Metabolic Syndrome (PREDIMED-Plus Cohort). Nutrients.

[47] Kofla-Dłubacz A., Pytrus T., Akutko K., Sputa-Grzegrzółka P., Piotrowska A., Dzięgiel P. (2022). Etiology of IBD—Is it still a mystery?. Int. J. Mol. Sci..

[48] Piovani D., Danese S., Peyrin-Biroulet L., Nikolopoulos G.K., Lytras T., Bonovas S. (2019). Environmental risk factors for inflammatory bowel diseases: An umbrella review of meta-analyses. Gastroenterology.

[49] Petrus R.R., do Amaral Sobral P.J., Tadini C.C., Gonçalves C.B. (2021). The NOVA classification system: A critical perspective in food science. Trends Food Sci. Technol..

[50] Tompa O., Kiss A., Soós S., Lakner Z., Raner A., Kasza G., Szakos D. (2025). Fifteen Years of NOVA Food-Processing Classification: “Friend or Foe” Among Sustainable Diet Indicators? A Scoping Review. Nutr. Rev..

[51] O’Connor L.E., Herrick K.A., Papier K. (2024). Handle with care: Challenges associated with ultra-processed foods research. Int. J. Epidemiol..

[52] Cordova R., Viallon V., Fontvieille E., Peruchet-Noray L., Jansana A., Wagner K.-H., Kyrø C., Tjønneland A., Katzke V., Bajracharya R. (2023). Consumption of ultra-processed foods and risk of multimorbidity of cancer and cardiometabolic diseases: A multinational cohort study. Lancet Reg. Health Eur..

[53] Mendoza K., Smith-Warner S.A., Rossato S.L., Khandpur N., Manson J.E., Qi L., Rimm E.B., Mukamal K.J., Willett W.C., Wang M. (2024). Ultra-processed foods and cardiovascular disease: Analysis of three large US prospective cohorts and a systematic review and meta-analysis of prospective cohort studies. Lancet Reg. Health Am..

[54] Martinez-Perez C., Daimiel L., Climent-Mainar C., Martínez-González M.Á., Salas-Salvadó J., Corella D., Schröder H., Martinez J.A., Alonso-Gómez Á.M., Wärnberg J. (2022). Integrative development of a short screening questionnaire of highly processed food consumption (sQ-HPF). Int. J. Behav. Nutr. Phys. Act..

[55] Benitez S.B., Sathar S., Forester S., Jennings-Dobbs E., Black R. (2025). WISEcode Ultra-Processed Food (Wc-UPF™): A data-driven precision tool for evaluating and categorizing processed foods. Curr. Dev. Nutr..

[56] Black R., Berciano S., Sathar S., Forester S., Reyes E., Li Z., Burton-Freeman B., Drewnowski A. (2025). Ultra-processed foods are not all alike: A novel, objective approach to differentiate among processed foods including those classified as NOVA 4. Curr. Dev. Nutr..

[57] Naimi S., Viennois E., Gewirtz A.T., Chassaing B. (2021). Direct Impact of Commonly Used Dietary Emulsifiers on Human Gut Microbiota. Microbiome.

[58] Trakman G.L., Lin W., Wilson-O’Brien A.L., Stanley A., Hamilton A.L., Tang W., Or L., Ching J., Morrison M., Yu J. (2020). Development and Validation of Surveys to Estimate Food Additive Intake. Nutrients.

[59] Tricco A.C., Lillie E., Zarin W., O’Brien K.K., Colquhoun H., Levac D., Moher D., Peters M.D., Horsley T., Weeks L. (2018). PRISMA Extension for Scoping Reviews (PRISMAScR): Checklist and Explanation. Ann. Intern. Med..

